# Response of leaf endophytic bacterial community to elevated CO_2_ at different growth stages of rice plant

**DOI:** 10.3389/fmicb.2015.00855

**Published:** 2015-08-31

**Authors:** Gaidi Ren, Huayong Zhang, Xiangui Lin, Jianguo Zhu, Zhongjun Jia

**Affiliations:** ^1^State Key Laboratory of Soil and Sustainable Agriculture, Institute of Soil Science – Chinese Academy of SciencesNanjing, China; ^2^Key Laboratory of Soil Environment and Pollution Remediation, Institute of Soil Science – Chinese Academy of SciencesNanjing, China

**Keywords:** free-air CO_2_ enrichment (FACE), growth stage, rice leaves, plant endophyte, bacterial community, 454 pyrosequencing

## Abstract

Plant endophytic bacteria play an important role in plant growth and health. In the context of climate change, the response of plant endophytic bacterial communities to elevated CO_2_ at different rice growing stages is poorly understood. Using 454 pyrosequencing, we investigated the response of leaf endophytic bacterial communities to elevated CO_2_ (eCO_2_) at the tillering, filling, and maturity stages of the rice plant under different nitrogen fertilization conditions [low nitrogen fertilization (LN) and high nitrogen fertilization (HN)]. The results revealed that the leaf endophytic bacterial community was dominated by Gammaproteobacteria-affiliated families, such as Enterobacteriaceae and Xanthomonadaceae, which represent 28.7–86.8% and 2.14–42.6% of the total sequence reads, respectively, at all tested growth stages. The difference in the bacterial community structure between the different growth stages was greater than the difference resulting from the CO_2_ and nitrogen fertilization treatments. The eCO_2_ effect on the bacterial communities differed greatly under different nitrogen application conditions and at different growth stages. Specifically, eCO_2_ revealed a significant effect on the community structure under both LN and HN levels at the tillering stage; however, the significant effect of eCO_2_ was only observed under HN, rather than under the LN condition at the filling stage; no significant effect of eCO_2_ on the community structure at both the LN and HN fertilization levels was found at the maturity stage. These results provide useful insights into the response of leaf endophytic bacterial communities to elevated CO_2_ across rice growth stages.

## Introduction

Just as animals have a complex microbiota, plants are normally colonized by diverse microorganisms rather than existing as axenic organisms (Jackson et al., 2013). It has been stated that a plant completely free of microorganisms does (almost) not exist, neither actually nor in the history of land plants (Partida-Martínez and Heil, 2011). Endophytic bacteria, which colonize the internal tissue of the plant, represent a widespread and ancient symbiosis (Partida-Martínez and Heil, 2011). They can influence host growth and function in many ways. Directly, they can contribute to plant growth by improving the availability of nutrients, such as phosphate (Quecine et al., 2012; Ji et al., 2014), by producing plant hormones, such as indole acetic acid (IAA; Quecine et al., 2012; Khan et al., 2014), and by fixing nitrogen (Knoth et al., 2013; Madhaiyan et al., 2013). Indirectly, they may improve the resistance of host plants to pathogens through increased competition for the same ecological niche (Loaces et al., 2011) or production of antimicrobial substances such as antibiotics (Sessitsch et al., 2004).

The identification of plant endophytic bacteria has largely been based on the cultivation-dependent method (Adhikari et al., 2001; Singh et al., 2006; Muthukumarasamy et al., 2007). However, only less than 1% of the microorganisms in the environment can be cultured because of unknown growth requirements of many microorganisms (Amann et al., 1995) or because some microbial cells are in viable but non-cultivable states (Tholozan et al., 1999). A few culture-independent studies have also been reported for the characterization of plant endophytic bacterial communities *via* 16S rRNA gene-based molecular techniques such as terminal restriction fragment length polymorphism (T-RFLP; Reiter et al., 2002; Manter et al., 2010; Ding et al., 2013; Thijs et al., 2014), the denaturing gradient gel electrophoresis (DGGE) approach (Araújo et al., 2002; Hardoim et al., 2011) and the clone library construction method (Jin et al., 2014). However, due to the relatively lower resolution of these techniques, the diversity of plant endophytic bacteria is far from being understood. The emergence of next-generation sequencing technology allows sequence efforts that are orders of magnitude greater than ever before and therefore have opened a new frontier in microbial diversity determination (Huse et al., 2008; Hollister et al., 2010). This technique has been used to evaluate the diversity of endophytic bacterial communities from the roots of rice (Zhang et al., 2013) and potato (Manter et al., 2010), and the leaves of rice (Ren et al., 2015) and commercial salad (e.g., baby spinach and lettuce; Jackson et al., 2013), and from the leaves and roots of *Arabidopsis thaliana* (Bodenhausen et al., 2013). This technique has also been used to determine the whole genome sequence of plant endophytic bacteria isolates (Zhu et al., 2012).

The rise in atmospheric CO_2_ concentration is projected to have a profound impact on the properties and functioning of the terrestrial ecosystem (Ainsworth and Long, 2005; Rosenzweig et al., 2007). A number of research studies have been conducted to understand the changes in plant physiology (Ainsworth et al., 2002; Bernacchi et al., 2003), plant growth and yield (Idso and Idso, 1994; Ainsworth et al., 2002; Belote et al., 2004), and plant community composition (Leadley et al., 1999; Belote et al., 2004) under the condition of elevated atmospheric CO_2_. However, within the context of climate changes, the response of the plant endophytic bacterial community, despite acting as a paramount player in plant growth and health and nutrient biogeochemical cycling, are poorly understood. The response of the plant endophytic bacterial community to elevated CO_2_ may be influenced by other environmental factors. Specifically, elevated CO_2_ often has been shown to have a stimulation effect on plant growth (Ainsworth and Long, 2005; Reich and Hobbie, 2013). However, the magnitude and sustainability of the CO_2_-enhanced plant biomass accumulation may be influenced by the nitrogen (N) level in the soil (Reich and Hobbie, 2013). A few studies (Kim et al., 2001; Anten et al., 2004; Reich et al., 2006) have shown that increased nitrogen levels in soil amplified the effect of elevated CO_2_ on plant productivity. Changes in plant growth may further lead to variations in the plant-associated bacterial community structure. Furthermore, over different growth stages of the host plant, the plant endophytic bacterial community is likely subject to dynamic changes over time.

Rice, a main source of nourishment for over 50% of the world’s population, is by far one of the most important staple food crops in the world (Gyaneshwar et al., 2001). The responses of the leaf endophytic bacterial communities from the rice plant to elevated CO_2_ under different nitrogen fertilization levels at the tillering, filling, and maturity stages was investigated in this study using the 16S rRNA gene-based 454 pyrosequencing technique. Our aim was to reach a deep understanding of the response of the leaf endophytic bacterial community to elevated CO_2_ at different nitrogen application levels over different rice growth periods.

## Materials and Methods

### Experimental Site Description

The FACE (free air CO_2_ enrichment) facility was set up in 2004 in Yangzhou, Jiangsu Province, China (119°42′0″E, 32°35′5″N). This area is a typical agricultural region for rice-wheat rotation in China. Rice planting has been ongoing for more than 50 years in this area. This region is also an important area for rice production in China. The relevant soil properties are as follows: soil bulk density 1.2 g cm^-3^, sand (2–0.02 mm) 57.8%, silt (0.02–0.002 mm) 28.5%, clay (<0.002 mm) 13.7%, and pH (H_2_O) 6.8. The climatic conditions are as follows: (1) mean annual precipitation temperature: 15°C; (2) mean annual precipitation: 900–1,000 mm; (3) average annual sunshine: approximately 2,132 h; and (4) annual frostless time: more than 220 days.

### FACE System

Details of the set-up and performance of the China rice-FACE facility have been described previously (Okada et al., 2001; Liu et al., 2002). Briefly, the FACE system consists of six plots distributed in different paddy fields having similar soil characteristics, nutrient levels (total nitrogen 1.45 g kg^-1^, total phosphorus 0.63 g kg^-1^, total potassium 14.0 g kg^-1^, available phosphorus 10.1 mg kg^-1^, available potassium 70.5 mg kg^-1^), and agronomic histories. Three plots were randomly allocated for the elevated CO_2_ treatments (hereinafter called eCO_2_) and three were maintained under ambient conditions (hereinafter referred to as aCO_2_). In the eCO_2_ plots, the rice plants were grown in octagonal “rings” with a diameter of 12 m. The target CO_2_ concentration in the center of the eCO_2_ rings was 200 ± 40 μmol mol^-1^ higher than that of the aCO_2_. To avoid treatment crossover, the eCO_2_ rings were separated from aCO_2_ plots by more than 90 m. Pure CO_2_ gas was sprayed from peripheral emission tubes, which were set 50–60 cm above the canopy. The CO_2_ concentration in the eCO_2_ rings was monitored and controlled by a computer program.

### Rice Cultivation

On June 20th, 2010, seedlings of japonica rice cultivar ‘Wuxiangjing 14,’ a major local cultivar with high-yield potential, were manually transplanted at a density of three seedlings per hill and 24 hills m^-2^ into the plots. Each ring was further partitioned into two identical subplots to test the impact of two nitrogen (N) fertilization levels, which were provided as urea (N, 46%) and a compound fertilizer [(N:P_2_O_5_:K_2_O = 15:15:15, %): low N (LN, 15 g N m^-2^) and high N (HN, 25 g N m^-2^)]. For all of the N fertilization levels, the phosphorus (P) and potassium (K) fertilizers were supplied at rates of 7 g P_2_O_5_ m^-2^ and 7 g K_2_O m^-2^, respectively. For the HN level, 36% of the N was applied as a basal dressing prior to transplanting (June 19th); 24% of the N was applied as a side dressing at early tillering (July 27th), and 40% of the N was provided at the panicle initiation (August 1st). For the LN fertilization level, 60% of the N was used as a basal dressing; the remainder (40% of the N) was applied at the panicle initiation as supplied in the HN fertilization level. For all of the N levels, P and K were both supplied as a basal dressing. To prevent treatment crossover, a 30-cm polyvinyl chloride (PVC) barrier was pushed 10 cm into the soil between the LN and HN subplots.

### Sample Collection

Leaf samples (more than 20 g) were collected on August 4th, September 16th, and October 26th, 2010 when the rice was at the tillering (when the tiller number is continently increasing continuously until it reaches to the point of maximum tillering), filling (when a milky white substance was accumulated in the grains and gradually became the texture of bread dough), and maturity (when the endosperm become hard and opaque, the dry matter of the grain becomes constant, and the grain turns golden brown) stages (Yoshida, 1981), respectively. The collected leaves from each subplot (a total of 12 subplots) were respectively bulked together, placed into sterile bags, put into an incubator (4°C), and brought to the lab. The laboratory analysis (i.e., DNA extraction) was conducted immediately after the sampling was finished at each time point. In total, 12 leaf samples were collected at each stage, and 36 leaf samples were obtained across all of the tested growth stages. The meteorological conditions, including the average daily air temperature, daily solar radiation, and daily precipitation during the sampling period, have been reported previously (Ren et al., 2014). When the tillering-, filling-, and maturity-leaf samples were collected, the average daily temperature was 31.2, 24.1, and 9.1°C, respectively; the mean daily solar radiation was 12.1, 13.7, and 5.14 MJ m^-2^, respectively; and the daily precipitation at all of the sampling points was 0 mm.

### Bacterial Cells Harvest and DNA Extraction

The collection of leaf endosphere bacteria was initiated after washing leaf-surface (i.e., phyllosphere) three times for bacteria, as previously described (Ren et al., 2015). Briefly, the leaves were immersed in sterile TE buffer (10 mM Tris-HCl, 1 mM EDTA, pH 8.0). Vigorous shaking at 250 rpm was then conducted to dislodge the microbial cells from the leaf surface. The dislodging procedure was repeated three times. Then, the collection of leaf endosphere bacterial cells was performed by submerging ground leaf samples in sterile Tris-EDTA (TE) buffer and washing three times. Similar to the collection method for the leaf-surface (i.e., phyllosphere) bacterial cells, three cycles of washing were performed to collect the leaf endophytic bacterial cells. The cell suspension resulting from each washing process was filtered through sterile glass wool to remove any visible large particles. The resulting three cell suspensions were pooled to maximize the recovery efficiency of the leaf endosphere bacterial cells. The combined cell suspension was then centrifuged, the supernatant was discarded, and the bacterial cell pellets were resuspended in 2 mL of TE buffer for further DNA extraction. Based on our preliminary experiment (Supplementary information), the majority of the phyllosphere bacterial cells were removed from the leaf surface after the three-time removal procedures because the DNA band from the fourth-collection of phyllosphere bacteria was greatly weaker (not visible) compared with that from the initial three-time-collection as revealed by the DNA electrophoresis results. For this reason, the dislodging of the phyllosphere bacterial cells was performed three times in the current study. Our preliminary experiment (Supplementary information) also suggested that the leaf endosphere bacteria would not suffer substantially heavy contamination by the phyllosphere bacteria after three times’ washing procedures for phyllosphere bacteria.

The collected cell suspension of the leaf endosphere bacteria was then used for DNA extraction, as previously reported (Garbeva et al., 2001; Araújo et al., 2002; Ren et al., 2015). In brief, three cycles of freezing with liquid nitrogen for 10 min and thawing at 65°C for 30 min was performed. A DNA-free lysozyme solution (40 μL of 10 mg mL^-1^) was added to enhance the cell lysis and the resulting solution was incubated at 37°C for 2 h. Then, 200 μL of a 20% sterilized sodium dodecyl sulfate (SDS) solution and 32 μL of a 20 mg mL^-1^ DNA-free proteinase K (Roche Applied Science) were added and the resulting mixture was incubated at 37°C overnight. Next, 800 μL of 5 mol L^-1^ sterilized NaCl was added, and the mixture was centrifuged. The supernatant was then purified using an equal volume of chloroform/isoamyl alcohol solution (24:1). The aqueous phase was then transferred. To precipitate the DNA pellets, 0.6 volumes of isopropanol were then added, and the mixture was incubated at 4°C for 30 min and centrifuged at 12,000 rpm for 15 min. Finally, the DNA pellet was washed with 70% pre-cooled ethanol, air dried, and re-dissolved in sterilized TE buffer. The whole cell collection and DNA extraction process was performed aseptically within an aseptic room.

### Pyrosequencing and Data Analysis

The 454 pyrosequencing was performed on a Roche 454 GS FLX Titanium pyrosequencing platform. Fusion primers consisting of adaptor A or B, key sequence, barcode and template specific sequences were used in this study. Specifically, the V4–V5 region of the bacterial 16S rRNA gene was amplified with the forward primer 515F [5′-CGTATCGCCTCCCTCGCGCCA+TCAG + (6 bp Barcode) + (GTGCCAGCMGCCGCGG)-3′] and the reverse primer 907R [5′-CTATGCGCCTTGCCAGCCCGC + TCAG + (6 bp Barcode) + (CCGTCAATTCMTTTRAGTTT)-3′]. The 50 μL PCR reaction mixture contained 1 × PCR buffer (Mg^2+^ plus), 0.2 mM of each deoxynucleoside triphosphate, 0.4 mM of each primer, 1.25 U of TaKaRa Taq HS polymerase (TaKaRa Biotech, Dalian, China), and 1 μL of DNA template. The PCR amplification program included initial denaturation at 94°C for 5 min, followed by 32 cycles of 94°C for 30 s, 55°C for 30 s, and 72°C for 45 s, and a final extension at 72°C for 5 min. The PCR products were subjected to electrophoresis using a 2.0% agarose gel. The amplicon band with a correct size (475 bp) was excised from the gel and then purified with an agarose gel DNA purification kit (TaKaRa Biotech, Dalian, China). The concentration of the cleaned PCR products was measured using the Qubit hs-DS-DNA kit (Invitrogen, Carlsbad, CA, USA) on a Tecan Infinite F200 Pro plate reader. All of the amplicons were then combined in equimolar amounts and sequenced on a Roche 454 GS FLX Titanium pyrosequencing machine (454 Life Science, Branford, CT, USA).

The pyrosequencing data analysis was processed using the Quantitative Insights into Microbial Ecology (QIIME) pipeline (http://qiime.org/). In brief, low quality sequences, which have lengths of <200 bp, an average quality score of <25, ambiguous nucleotides of >0, homopolymer >6, and primer mismatches were trimmed and the barcodes were determined to assign sequence reads to the proper samples. Then, the chimeras were detected using the UCHIME algorithm based on a database of chimera-free sequences (Edgar et al., 2011). The sequences, which were assigned to a mitochondrial or chloroplast origin were eliminated with the Metaxa software tool (Bengtsson et al., 2011) and the V4–V5 region was extracted with the V-Xtractor software tool (Hartmann et al., 2010). The operational taxonomic units (OTUs) with a 97% sequence similarity were then generated using the uclust clustering method (Edgar, 2010). The phylogenetic affiliation of OTUs was analyzed by the Ribosomal Database Project classifier (Wang et al., 2007). The pyrosequencing data have been deposited in the DNA Data Bank of Japan (DDBJ) under accession numbers DRA001097 and DRA001098.

Due to technical reasons, the sequence depth obtained for each sample differed (Aguirre de Cárcer et al., 2011). In this study, the range of valid sequence depth was 2,865–9,650 sequences for each sample after applying all of the quality filters. To standardize the number of sequences per sample, each community should be rarefied to a common sequence depth (Aguirre de Cárcer et al., 2011; Fierer et al., 2011). In this study, each community was rarefied to 2,800 sequences before further analysis, at which sequence depth the sequence coverage averaged 87.2% determined by the good’s coverage: 1- f1/n, where f1 is the number of singletons (OTUs each represented by one sequence) and n denotes the sequence size (2,800 sequence here; Good, 1953). This observed coverage value suggests that the majority of OTUs had been captured. The rarefaction procedure was repeated 20 times on the sample-by-OTU table. The Bray–Curtis distance, due to its robustness (Faith et al., 1987), was then used to measure the dissimilarity based on the rarefied OTU table. The mean was taken over the 20 distance matrices and used for subsequent analysis. A principal coordinates analysis (PCoA) of the Bray–Curtis distance was performed to determine the change in the community structure. Three different non-parametric analysis methods, including analysis of similarities (ANOSIM), non-parametric multivariate analysis of variance using distance matrices (adonis), and a multi-response permutation procedure (MRPP), was used to examine whether there were significant differences in community structures among the treatments. The Bray–Curtis distance was used for the ANOSIM, adonis, and MRPP analyses. PCoA, ANOSIM, adonis, and MRPP were processed with the “vegan” package in R software version 3.1.2.

## Results

### Overall Taxonomic Distribution

**FIGURE 1:**
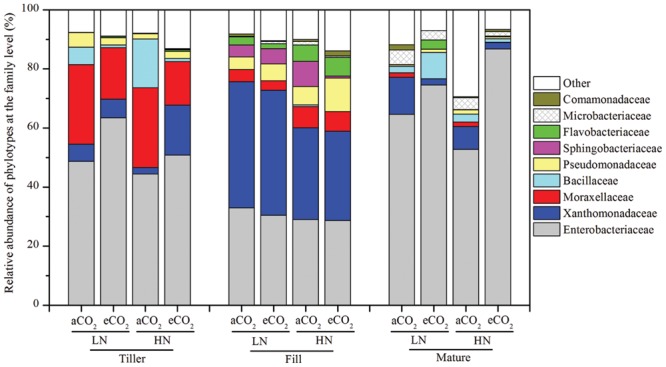
**The relative abundance of the leaf endophytic bacterial phylotypes at the family level.** The designations Tiller, Fill, and Mature indicate that the rice leaves were sampled at the tillering, filling, and maturity stages, respectively. The aCO_2_ and eCO_2_ represent the treatments of ambient and elevated CO_2_, respectively. The LN and HN refer to low and high levels of nitrogen fertilization, respectively. The statistical analysis results are the same as those in **Figure 4**.

In total, 210,423 valid sequence reads were recovered across all 36 samples (an average of 5,845 sequences per sample) after applying all of the quality filters (Supplementary Table S1). Given that ≥91.1% of sequence reads could be classified taxonomically at the family or higher taxonomic level, but only 49.4% of sequences could be assigned with a taxonomic identity at the genus level (Supplementary Table S1), the taxonomic distribution of the leaf endophytic bacterial community was then examined at the family level. The results indicated that the leaf endophytic bacterial community was primarily made up of nine families (**Figure 1**) covering five phyla/classes: Gammaproteobacteria, Firmicutes, Bacteroidetes, Actinobacteria, and Betaproteobacteria. Of the nine families, the most heavily sequenced populations were the Gammaproteobacteria-affiliated families. For example, the relative abundances of two of the Gammaproteobacteria-affiliated families, Enterobacteriaceae and Xanthomonadaceae, reached 28.7–86.8% and 2.14–42.6%, respectively, at all of the tested growth stages (**Figure 1**); another two Gammaproteobacteria-affiliated families, Moraxellaceae and Pseudomonadaceae, were also abundantly detected at both the tillering and the filling stages, with a relative abundance of 3.20–27.1% for Moraxellaceae and 1.80–11.4% for Pseudomonadaceae (**Figure 1**). The Firmicutes-affiliated family, Bacillaceae, was abundantly present at the tillering and maturity stages; the Bacteroidetes-affiliated families, Sphingobacteriaceae and Flavobacteriaceae, were abundant at the filling stage; and the Microbacteriaceae, belonging to Actinobacteria, was abundantly found at the maturity stage (**Figure 1**).

### Bacterial Diversity

**FIGURE 2:**
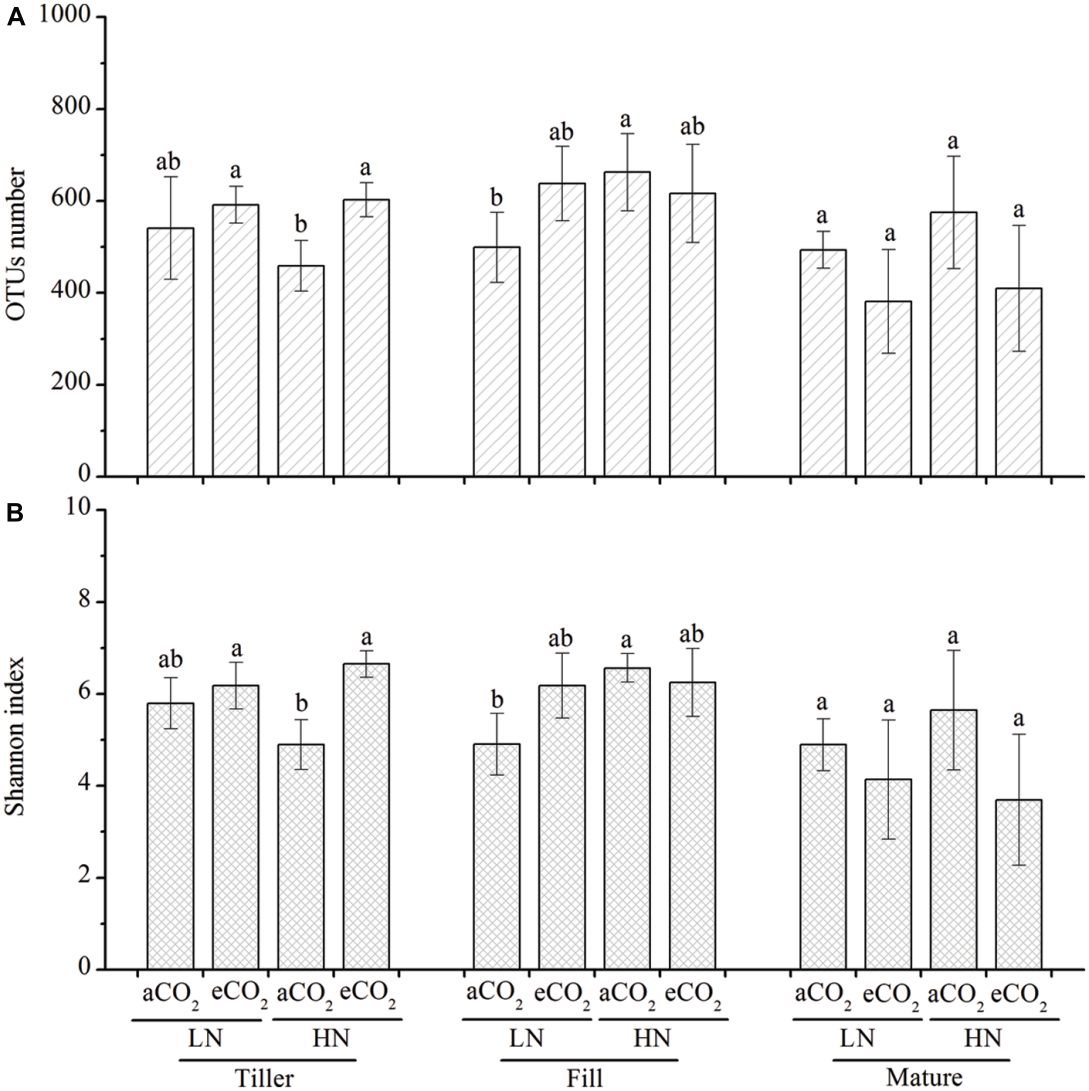
**The OTUs number (A) and Shannon index (B).** The OTUs number and Shannon index were obtained by using 2,800 subsampled sequence reads from each community. The different letters represent significant differences (*P* < 0.05; Duncan’s multiple range test). All other designations are the same as those in **Figure 1**.

The observed OTUs (**Figure 2A**) and the Shannon index (**Figure 2B**) was used to compare the bacterial richness and diversity, respectively, based on subsampled same number of sequences (2,800 sequences per sample, at which sequence depth the majority of the OTUs have been captured based on the mean sequence coverage (87.2%), which was determined by the good’s coverage). We did not observe consistent changes that could be attributed to eCO_2_, N fertilization, or different growth stages. For example, eCO_2_ significantly (*P* < 0.05) increased the bacterial richness (**Figure 2A**) and diversity (**Figure 2B**) under high N fertilization (HN) at the tillering stage, whereas no significant effect was observed at this stage under the low N fertilization (LN) treatment and at other growth stages under the LN and HN treatments. Enhanced N fertilization significantly (*P* < 0.05) increased the bacterial richness (**Figure 2A**) and diversity (**Figure 2B**) under ambient CO_2_ treatment at the filling stage but it did not show significant influence under eCO_2_ condition at this stage. In addition, no significant differences between LN and HN were observed at the tillering or maturity stage.

### Bacterial Community Structure

**FIGURE 3:**
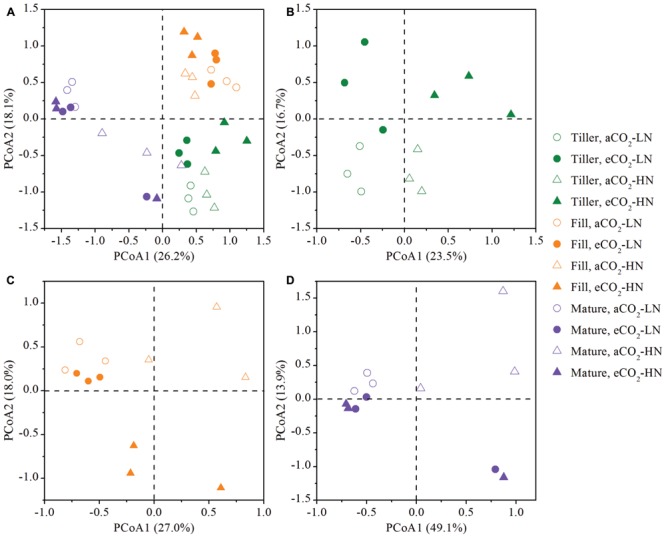
**Principal coordinates analysis (PCoA) of microbial communities from all samples (A) or those samples collected at the tillering (B), filling (C), and maturity (D) stages, respectively.** The percentage in parentheses denotes the proportion of variation explained by each ordination axis. All other designations are the same as those in **Figure 1**.

To examine the shift of the leaf endophytic bacterial community structures in response to eCO_2_ under different N fertilization levels at the tillering, filling, and maturity stages, a PCoA was performed based on the 454 pyrosequencing data (**Figure 3**). First, to understand how all of the samples were related to each other, a global PCoA was performed on the same ordination plot (**Figure 3A**). The results revealed that the axis separated the samples based on the growth stage, and the difference in bacterial communities from different treatment (CO_2_ and N treatments) was less than the difference between the different growth stages. Three non-parametric multivariate analyses (adonis, ANOSIM, and MRPP) also illustrated significant differences (*P* < 0.05) between the growth stages in the bacterial community structure (**Table 1**), whereas the significant difference was not always observed when the CO_2_ and N effects was tested by the three non-parametric multivariate analyses (**Table 1**). To further understand how bacterial communities from the same growth stage related to one another, the PCoA of the bacterial communities derived from the tillering, filling, and maturity stages were processed on three individual ordination plots (**Figures 3B–D**). The results showed that the magnitude of the eCO_2_ effect on the bacterial community structure varied greatly under different N fertilization levels and at different growth stages. The eCO_2_ showed a significant effect on the community structure under both LN and HN levels at the tillering stage. Specifically, at the tillering stage, the samples from aCO_2_ and eCO_2_ samples were distributed in different parts of the PCoA data space under both LN and HN levels, and adonis, ANOSIM, and MRPP further showed significant differences (*P* < 0.05) between bacterial communities at aCO_2_ and eCO_2_ (**Table 1**). However, a significant effect of eCO_2_ on the bacterial community structure was only observed under HN, rather than under the LN condition at the filling stage (**Table 1**). The PCoA result also revealed that the aCO_2_ and eCO_2_ samples under the HN level were distributed distantly in the data space at the filling stage, whereas those samples from aCO_2_ and eCO_2_ under the LN condition clustered closely (**Figure 3C**). At the maturity stage, no significant effect of eCO_2_ on the community structure under both LN and HN fertilization levels were observed (**Table 1**), although most of aCO_2_ and eCO_2_ samples were distributed in a different data space (**Figure 3D**).

**Table 1:** Significance tests using three statistical approaches to assess the effects of growth stages, CO_2_, and N fertilization on the overall microbial community structure.

Compared groups	Adonis^a^		ANOSIM^b^		MRPP^c^	
	*F*	*P* ^d^	*R*	*P* ^d^	*δ*	*P* ^d^
**Global test**	2.226	0.001	0.463	0.001	0.634	0.001
**Stage effect**						
Till vs. Fill	5.423	**0.003**	0.630	**0.003**	0.628	**0.003**
Till vs. Maturity	5.474	**0.003**	0.510	**0.003**	0.690	**0.003**
Fill vs. Maturity	8.275	**0.003**	0.642	**0.003**	0.648	**0.003**
**CO_2_ effect**						
aCO_2_ vs. eCO_2_ under Tiller-LN condition	1.697	**0.045**	0.500	**0.047**	0.507	**0.049**
aCO_2_ vs. eCO_2_ under Tiller-HN condition	1.405	**0.046**	0.296	**0.046**	0.680	**0.047**
aCO_2_ vs. eCO_2_ under Fill-LN condition	1.309	0.280	0.250	0.281	0.472	0.105
aCO_2_ vs. eCO_2_ under Fill-HN condition	1.807	**0.043**	0.556	**0.042**	0.518	**0.045**
aCO_2_ vs. eCO_2_ under Mature-LN condition	0.777	0.482	-0.167	0.701	0.773	0.482
aCO_2_ vs. eCO_2_ under Mature-HN condition	0.593	0.792	-0.167	0.899	0.688	0.605
**N fertilization effect**						
LN vs. HN under Tiller-aCO_2_ condition	1.987	**0.043**	0.250	**0.045**	0.608	**0.044**
LN vs. HN under Tiller-eCO_2_ condition	2.089	**0.041**	0.482	**0.043**	0.595	**0.042**
LN vs. HN under Fill-aCO_2_ condition	1.819	0.108	0.583	0.119	0.475	0.109
LN vs. HN under Fill-eCO_2_ condition	1.995	**0.042**	0.333	**0.041**	0.516	**0.044**
LN vs. HN under Mature-aCO_2_ condition	0.643	0.704	-0.250	0.804	0.841	0.714
LN vs. HN under Mature-eCO_2_ condition	0.322	0.795	-0.222	0.891	0.701	0.723

*All of the tests (adonis, ANOSIM, and MRPP) are non-parametric multivariate statistical analyses based on dissimilarities among samples.*

*^a^Permutational multivariate analysis of variance using distance matrices. The significance was examined by F-tests based on sequential sums of squares from permutations of the raw data.*

*^b^Analysis of similarities. R was obtained based on the difference of the average ranks between groups and within groups. The significance of R was tested by permuting the grouping vector to obtain the empirical distribution of R under the null-model.*

*^c^Multi-response permutation procedure. The statistic δ indicates the overall weighted mean of the within-group means of the pairwise dissimilarities among the sampling units. The significance test indicates the fraction of permuted δ that is less than the observed δ.*

*^d^A Bonferroni correction for the P-value was applied with the exception of the global test.*

*Bold numbers indicate significance at less than 0.05 level. All other designations are the same as those in **Figure 1**.*

### Specific Bacterial Populations in Different Treatments

Given that 91.1% of the sequences could be classified at the family level, but only 49.4% of sequences could be classified at the genus level (Supplementary Table S1), specific bacterial phylotypes were then analyzed at the family level. The relative abundance shifts of the main families in the different treatments are shown in **Figure 1**. The leaf samples at the same growth stage harbored a similar community composition and the endophytic bacterial communities sampled throughout the growth period were greatly different from one another. For instance, the family Enterobacteriaceae showed a great variations in the relative abundance at different growth stages: they comprised as high as 44.4–63.5% of the entire bacterial community at the tillering stage, and then were reduced to 28.7–33.0% at the filling stage and became dominant again (accounting for 52.5–86.8% of the total sequence reads) at the maturity stage (**Figure 1**). The family Xanthomonadaceae was abundantly found at the filling stage and comprised 30.2–42.6% of total bacterial community, whereas they had a relatively great lower relative abundance at the tillering and maturity stages, only making up of 2.18–16.8% and 2.14–12.5% of the total sequence reads, respectively (**Figure 1**). Similarly, the family Moraxellaceae showed a high relative abundance at the tillering stage (14.8–26.9%), whereas only 3.20–6.65% and 0–1.55% were found at the filling and maturity stages, respectively. The family Bacillaceae were abundant at the tillering (0.98–16.5%) and maturity (1.21–8.94%) stages but were rarely detected at the filling stage (0–0.58%). Additionally, the relative abundance of Pseudomonadaceae were exclusively high at the tillering (1.77–4.93%) and filling (4.29–11.4%) stages rather than at the maturity stage (only 0.53–1.42%). Another stage-specific phylotype was the family Sphingobacteriaceae, which was only abundantly found only at the filling stage (0.61–4.07%) rather than at the tillering (0–0.05%) and maturity (0–0.15%) stages. Likewise, the family Flavobacteriaceae was exclusively abundant at the filling and maturity stages and the two families, Microbacteriaceae and Comamonadaceae, were abundantly detected at the filling and maturity stages. These results revealed a stage-specific bacterial community composition of the rice leaves.

**FIGURE 4:**
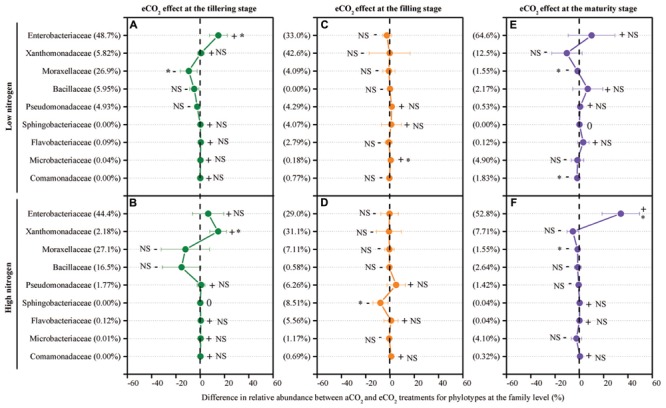
**The effect of eCO_2_ on the relative abundance of leaf endophytic bacterial phylotypes at the family level at the tillering (A,B), filling (C,D), and maturity (E,F) stages.** The eCO_2_ effect was examined using the net difference in the relative abundance of bacterial taxa between the eCO_2_ and aCO_2_. The net difference in the relative abundance was calculated as the relative abundance under eCO_2_ minus the relative abundance of the phylotype under aCO_2_ at each N treatment level. The percentage value in the bracket refers to the relative abundance at aCO_2_. The error bar denotes the standard error of the mean. The phylotypes are presented in generally descending order based on their relative abundance in the aCO_2_ control treatment. The symbols “+”, “-”, and “0” indicate that the relative abundance was increased, decreased, or stable compared with the aCO_2_ control treatment. The symbol “^∗^” represents significant differences at *P* < 0.05, and NS represents no significant difference (*P* > 0.05). All other designations are the same as those in **Figure 1**.

The response of the leaf endophytic bacterial taxa to eCO_2_ under different N fertilization levels was examined. To examine which bacterial taxa responded to eCO_2_ and how they responded, the net difference in relative abundance of each family between eCO_2_ and aCO_2_ was determined to assess the shift of leaf endophytic bacteria in response to eCO_2_ (**Figure 4**). Overall, within those nine main bacterial populations, six families, Enterobacteriaceae, Xanthomonadaceae, Moraxellaceae, Sphingobacteriaceae, Microbacteriaceae, and Comamonadaceae, showed significant differences in relative abundance between the aCO_2_ and eCO_2_ treatments under the LN or HN condition at one of the tested growth stages (**Figure 4**). Of them, the family Moraxellaceae was significantly (*P* < 0.05) decreased in relative abundance due to the eCO_2_ treatment under the LN condition at the tillering stage (**Figure 4A**) and under both LN and HN conditions at the maturity stage (**Figures 4E,F**). The relative abundance of this phylotype also showed a slightly non-significant decrease by eCO_2_ under both the LN and HN conditions at the filling stage (**Figures 4C,D**). As for the other five significantly affected families, the changing direction of the relative abundance for a specific phylotype was not consistent (i.e., increase or decrease) in response to eCO_2_ at different growth stages and under different N fertilization conditions. For example, the most dominant and shared phylotype, Enterobacteriaceae, was significantly increased (*P* < 0.05) by eCO_2_ in terms of the relative abundance under the LN condition at the tillering stage (**Figure 4A**) and under the HN condition at the maturity stage (**Figure 4F**), whereas a slight decrease was observed at the filling stage (**Figures 4C,D**). The family Xanthomonadaceae significantly increased in relative abundance under the HN condition at the tillering stage (**Figure 4A**) whereas a prominent decrease was found under both the LN and HN conditions at the maturity stage (**Figures 1** and **4E,F**). Similarly, when exposed to eCO_2_, no consistent changing direction was found for these three families Sphingobacteriaceae, Microbacteriaceae, and Comamonadaceae along the growth stages (**Figure 4**).

## Discussion

The plant endophytic bacteria from different parts of the rice plant such as roots, stems, leaves, and seeds have been partially characterized by culture-dependent approach, clone library, T-RFLP, and metagenome methods. This study made an in-depth (an average of 5,845 sequences per samples) exploration of the leaf endophytic bacterial community using 454 pyrosequencing. Gammaproteobacteria-affiliated families, members of which have been reported to play a critical role in plant growth (Taghavi et al., 2009; Kim et al., 2012; Witzel et al., 2012), were the most heavily sequenced phylotypes in our study. The predominance of Gammaproteobacteria in the rice leaf endosphere was similar with a reported culture-dependent study showing that members of Gammaproteobacteria, *Pantoea* sp. and *Pseudomonas* sp. made up 51% of the entire leaf endophytic bacterial communities in three detected rice varieties (Ferrando et al., 2012). Another study also found the dominance of the Gammaproteobacteria-affiliated genus, *Pantoea*, in the leaf endosphere of the rice plant (Loaces et al., 2011). In the same paddy field, our previous study has revealed the prevalence of Gammaproteobacteria on the leaf surface (Ren et al., 2014). These two studies indicate that, albeit in different proportions, Gammaproteobacteria commonly and abundantly colonized both the leaf surface and the leaf endosphere of the rice plant. The finding that bacteria taxa were in both the internal and external parts of the rice plant, which was a finding similar to previous studies (Mano et al., 2006, 2007), suggests that the colonizer may come from a similar source. Plant endophytic bacteria have been considered to originate from the external environment and enter the plant through the stomata, lenticles, wounds, areas of emergence of lateral roots and germinating radicles (Mano and Morisaki, 2008). First, the internal bacteria of the leaf in our study may come from rain splashing off the soil since the rice leaves were close to the ground when the rice plant was young. As the plant grows, the bacteria could colonize the expanding leaves. Conversely, the wind and rain, thought to be a source of bacteria residing on the leaf surface (Bodenhausen et al., 2013), could also bring bacteria to the soil and hence to the plant endosphere as the plant grows. Second, the leaf external bacteria could also enter the internal tissue of leaves as endophytes. This may also be an explanation for the common colonization of both the leaf surface and the leaf endosphere. A third explanation is that those bacteria in the seeds or soil may enter the aboveground part including expanding leaves as the plant grows and develops through infecting seeds, the seedling after germination, or the emergence of lateral roots.

In this study, the strongest differences in the bacterial community composition and structure were observed on rice plants of different developmental stages. Conspicuously different microbial community structure in different growth periods was also observed in other plant species such as sugar beets (*Beta vulgaris* L.; Shi et al., 2014) and weeds (*Stellera chamaejasme* L.; Jin et al., 2013). Additionally, in our study, the leaves sampled in the same growth period had similar endophytic community compositions, whereas some bacterial populations were only abundantly detected at specific growth stages. Hence, it is hypothesized that the composition and distribution of leaf endophytic bacterial communities across different growth stages were not random, as was the case in leaf epiphytic bacterial communities along the growth period (Redford and Fierer, 2009). The observed temporal variability in the structure and composition of bacterial communities may be explained, in part, by variations in abiotic factors, such as climate conditions, although we cannot state exactly that the shift of a certain bacterial population is caused by specific climate factors. For instance, between the highest and the lowest average daily temperature, the difference was as high as 22.1°C (31.2, 24.1, and 9.1°C at the tillering, filling, and maturity sampling time point, respectively), and the solar radiation also varied greatly between three sampling time points (12.11, 13.86, and 5.14 MJ m^-2^ day^-1^, respectively). To some extent, our speculation was supported by previous studies that showed that the composition and fluctuation of endophytic bacterial communities from grapevine leaves was closely related to seasonality (Bulgari et al., 2014). Distinctly seasonal changes of leaf-associated bacterial communities have also been observed on trees (*Magnolia grandiflora*; Jackson and Denney, 2011). Moreover, the growth stage of plants not only includes changes in climate (water, temperature, and ultraviolet radiation) but also in plant host attributes (photosynthesis, respiration, stomatal conductance, etc.) and hence resource supplies (water, nutrients). Therefore, the temporal variability observed in this study in the structure and composition of leaf endophytic bacterial communities can be the result of a feedback to the host growth stage *per se* and to season (Peñuelas et al., 2012) or to both. The observation that several unique bacterial taxa were exceptionally abundant at specific stages may be due to the adaptive selection driven by changes in host physiology and by variations in the seasonal environment (Ellis et al., 1999; Hartmann et al., 2009). Further work is needed to identify the driving factors that led to the observed temporal shifts in the stage-specific bacterial populations, to test whether the seasonal pattern was repeatable from year to year, and whether the structure and composition of the leaf endophytic bacterial communities could follow a predictable successional pattern. In particular, to differentiate the contribution of the seasonal effect from the host physiology, it would be valuable to conduct a metabolomics study to investigate rice metabolites at different growth stages and determine their correlation to growth-stage related changes of the endophytes. Alternatively, conducting a controlled parallel experiment (e.g., using a greenhouse to make the plant grow under controlled environmental conditions) to the field experiment would also be helpful to sort out the confounding factors.

The eCO_2_ could also be an important factor leading to the shift in the leaf endophytic bacterial communities. For example, the significant influence of eCO_2_ on the community structure was observed under both the LN and HN levels at the tillering stage. At the specific bacterial population level, the relative abundance of the families Enterobacteriaceae, Xanthomonadaceae, Moraxellaceae, Sphingobacteriaceae, Microbacteriaceae, and Comamonadaceae was significantly affected by eCO_2_ under the LN or HN condition at one of the tested growth stages. We also observed a significant eCO_2_ effect on some of the abundant phylotypes at a finer taxonomic level, i.e., genus level (Supplementary Figures S1 and S2). Given that the primers are not generally available for each bacteria taxon, the relative abundance (instead of the absolute quantitative data of each bacterial population) was used in this study to elucidate the response patterns of each bacterial phylotype. The importance of eCO_2_ in structuring the microbial community in soil has been demonstrated in various ecosystems (Montealegre et al., 2000; Drissner et al., 2007; He et al., 2010). Here, we provided evidence that eCO_2_ could also influence the structure and the composition of plant-associated bacterial communities such as the leaf endophytic bacterial communities.

The eCO_2_ effect varied greatly under different N fertilization conditions and at the different growth stages. For example, at the tillering stage, we observed a significant influence of eCO_2_ on the community structure regardless of the N fertilization levels, suggesting a nitrogen-independent response, but a different case was found at the filling stage when the significant impact of eCO_2_ was observed under enhanced N fertilization rather than under the LN condition. This suggests a nitrogen fertilization-dependent response to eCO_2_ at the filling stage. Therefore, our results provided evidence for the necessity of incorporating the N fertilization practice and plant growth period when evaluating the biosphere response to elevated CO_2_ in the context of climate change. Previous studies of the impact of eCO_2_ on the structure of soil microbial communities have shown that the structure of the soil microbial communities were either stable (Austin et al., 2009; Ge et al., 2010) or changed under the eCO_2_ condition in various ecosystems (Montealegre et al., 2000; Drissner et al., 2007; He et al., 2010). The influence of eCO_2_ on microbial communities in soil could depend on various factors, and sampling time and N level should be considered as two of the most important factors in these studies.

A previous rice-FACE study also showed that the N level influenced the eCO_2_ effect on the leaf-associated bacterial community structure (Ikeda et al., 2015), which was similar to our finding that the response to eCO_2_ could be nitrogen-dependent at a certain growth stage. In contrast to our results, Ikeda et al. (2015) found that the most prominent effect of eCO_2_ was observed under a no N fertilization condition rather than under applied N fertilization (8 g m^-2^). In our study, a significant impact of eCO_2_ was observed under enhanced N fertilization (25 g m^-2^) rather than under the LN condition. The difference in growth period may be one of the reasons that lead to these discrepancies (Bulgari et al., 2014). In the Ikeda et al. (2015) study, the research was conducted when the rice plant was at the panicle initiation stage, whereas the significant eCO_2_ effect was observed at the tilling and filling stages in our study. Additionally, there was a difference in the rice varieties used in the Ikeda et al. (2015; *Oryza sativa* L. cv. Koshihikari) study and our study (‘Wuxiangjing 14’ japonica rice cultivar), which may also be one of the causal factors (Whipps et al., 2008; Müller et al., 2015).

In summary, culture-independent analysis using 454 pyrosequencing indicated that rice leaves harbored diverse endophytic bacterial phylotypes. The Gammaproteobacteria, which have been reported to play a critical role in plant growth, were found to be the most dominant population in this study. The most evident difference in the community structure and composition were observed on plants of different growth stages. Additionally, eCO_2_ changed the community structure at certain time points (tillering stage) or under certain N application conditions (HN at the filling stage). The alteration of the composition and structure of leaf endophytic bacterial communities is highly likely to have functional consequences given the versatile functions of plant endophytic bacteria. Further studies should be focused on exploring and testifying the potential roles, such as the functions that are relevant to global N cycling, plant health, and plant growth promotion, of particular phylotypes (e.g., Gammaproteobacteria affiliated families).

## Conflict of Interest Statement

The authors declare that the research was conducted in the absence of any commercial or financial relationships that could be construed as a potential conflict of interest.
