# Supplementary material for: Response of leaf endophytic bacterial community to elevated CO2 at different growth stages of rice plant
Source: Front Microbiol. 2015 Aug 31;6:855. doi: 10.3389/fmicb.2015.00855 (PMC4553393; doi:10.3389/fmicb.2015.00855)
Supplement: Supplementary file 1 [file Image_1.PDF]

Supplementary Material

Response of leaf endophytic bacterial community to elevated CO_2 at different growth stages of rice plant

Gaidi Ren[1,2], Huayong Zhang[1], Xiangui Lin[1], Jianguo Zhu[1], Zhongjun Jia[1*]

[1] State Key Laboratory of Soil and Sustainable Agriculture, Institute of Soil Science, Chinese Academy of Sciences, Nanjing, China

[2] Key Laboratory of Soil Environment and Pollution Remediation, Institute of Soil Science, Chinese Academy of Sciences, Nanjing, China

*** Correspondence:** Zhongjun Jia, State Key Laboratory of Soil and Sustainable Agriculture, Institute of Soil Science, Chinese Academy of Sciences, East Beijing Road No. 71, Nanjing, Jiangsu Province, 210008, China

jia@issas. ac.cn

This file includes:

Supplementary materials and methods

Supplementary Table S1

Supplementary Figure S1, S2, and S3

Supplementary materials and methods

Preliminary experiments of DNA extraction from the leaf endosphere and phyllosphere

The leaf samples were randomly selected for DNA extraction in this preliminary experiment. The leaf endosphere and phyllosphere (i.e., leaf-surface) bacterial cells were collected before the extraction of microbial genomic DNA. The collection of phyllosphere bacterial cells was performed as previously described (Delmotte et al., 2009; Redford and Fierer, 2009), with slight modifications. Specifically, the leaves were immersed in sterile TE buffer (10 mM Tris-HCl, 1 mM EDTA, pH 8.0). Vigorous shaking at 250 rpm was then conducted to remove the bacterial cells from the leaf surface. The resulting cell suspension was filtered through sterile glass wool to remove any visible large particles. The successive removal of the bacterial cells was repeated four times by adding TE buffer and filtering through the glass wool. The cell suspensions collected in the initial three washes were pooled (this suspension is referred to as the 3-time-suspension hereafter). The cell suspension collected in the 4th washing procedure is referred to as the 4th-suspension hereafter. Then, the 3-time-suspension and 4th-suspension were centrifuged for 10 min at 10,000 rpm, the supernatant was discarded and the pellets were resuspended in 2 mL of sterile TE buffer for further DNA extraction. The collection of bacterial cells from the leaf endosphere was conducted as previously reported (Garbeva et al., 2001; Araújo et al., 2002), with slight modifications. In brief, after harvest of the phyllosphere bacterial cells, the leaf samples were ground and immersed in sterile TE buffer. Then, similar to the collection method for the phyllosphere bacterial cells, the washing procedure was repeated four times. A 3-time-suspension and 4th-suspension were obtained and used for further DNA extraction.

The collected bacterial cell suspensions were then used for DNA extraction as previously described (Garbeva et al., 2001; Araújo et al., 2002), with slight modifications. Three cycles of freezing in liquid nitrogen for 10 min and thawing at 65°C for 30 min were conducted. Then, 40 μL of 10 $mg.mL^{-1}$ lysozyme was added to enhance the cell lysis. The resulting solution was incubated at 37°C for 2 h. Then, 200 μL of 20% sterilized sodium dodecyl sulfate (SDS) solution and 32 μL of 20 $mg.mL^{-1}$ DNA-free proteinase K (Roche Applied Science) were added. The resulting solution was incubated at 37°C overnight. A total of 800 μL of 5 $mol.L^{-1}$ sterilized NaCl solution was added, and the mixture was centrifuged. The resulting supernatant was then purified using an equal volume of a chloroform/isoamyl alcohol solution (24:1). The upper aqueous phase was transferred to a new tube. To precipitate the DNA pellets, 0.6 volumes of isopropanol was added to the new tube, and the mixture was incubated at 4°C for 30 min and centrifuged at 12,000 rpm for 15 min. The DNA pellets were then washed in 70% pre-cooled ethanol, air-dried, and re-dissolved in sterile TE buffer. The whole cell collection and DNA extraction process was

performed aseptically within an aseptic room.

DNA electrophoresis

The agarose gel electrophoresis method was applied to check the DNA quality (Figure S3). A total of 2.5 μl of the extracted DNA was loaded onto the gel. The DNA band from the 4th-suspension was greatly weaker (not visible) compared with that from the initial 3-time-suspension, indicating that the majority of the bacterial DNA had been extracted after the 3-time collection of bacterial cells. These results also revealed that if the collection of leaf endosphere bacteria was initiated after the three times' collection of phyllosphere bacteria, then the leaf endosphere bacteria would not suffer substantially heavy contamination by the phyllosphere bacteria.

References

Araújo, W.L., Marcon, J., Maccheroni, W., Van Elsas, J.D., Van Vuurde, J.W.L., and Azevedo, J.L. (2002). Diversity of endophytic bacterial populations and their interaction with *Xylella fastidiosa* in citrus plants. *Appl. Environ. Microbiol.* 68, 4906-4914. doi: 10.1128/aem.68.10.4906-4914.2002.

Delmotte, N., Knief, C., Chaffron, S., Innerebner, G., Roschitzki, B., Schlapbach, R., et al (2009). Community proteogenomics reveals insights into the physiology of phyllosphere bacteria. *Proc. Natl. Acad. Sci. U.S.A.* 106, 16428–16433. doi: 10.1073/pnas.0905240106.

Garbeva, P., Van Overbeek, L., Van Vuurde, J., and Van Elsas, J. (2001). Analysis of endophytic bacterial communities of potato by plating and denaturing gradient gel electrophoresis (DGGE) of 16S rDNA based PCR fragments. *Microb. Ecol.* 41, 369-383. doi: 10.1007/s002480000096.

Redford, A.J., and Fierer, N. (2009). Bacterial succession on the leaf surface: a novel system for studying successional dynamics. *Microb. Ecol.* 58, 189-198. doi: 10.1007/s00248-009-9495-y.

Supplementary Table

Table S1. Reads number and the percentage of sequences identified at different taxonomic levels.

Sample (growth stage[a], treatment[b])	High quality reads No.	Percentage of sequences identified at different taxonomic levels (%)				
		Phylum	Class	Order	Family	Genus
Tiller, aCO_2-LN	5,666±3,231	98.3±1.1	98.1±1.0	93.8±4.0	93.5±4.1	37.3±32.4
Tiller, eCO_2-LN	6,723±618	99.3±0.3	99.0±0.3	92.2±1.9	91.8±2.2	32.0±8.9
Tiller, aCO_2-NN	4,800±535	97.8±1.5	97.6±1.6	93.1±1.5	92.5±1.2	49.5±8.8
Tiller, eCO_2-NN	4,846±584	99.2±0.7	98.9±0.6	92.2±2.1	91.0±2.2	53.8±19.6
Fill, aCO_2-LN	6,801±1,935	99.6±0.2	98.9±0.7	93.6±3.1	93.0±3.5	36.3±28.3
Fill, eCO_2-LN	5,793±2,034	99.1±0.3	98.1±0.5	91.6±0.4	90.9±0.4	57.5±3.4
Fill, aCO_2-NN	5,705±1,197	99.1±0.4	98.1±0.6	92.2±1.3	91.1±2.0	57.2±8.6
Fill, eCO_2-NN	7,990±1,976	98.6±0.9	97.6±1.0	90.1±2.0	88.4±2.7	54.3±8.1
Mature, aCO_2-LN	4,999±2,185	99.0±1.3	97.7±1.6	95.3±1.6	91.9±5.2	38.6±25.7
Mature, eCO_2-LN	6,255±1,830	98.1±2.1	97.4±2.8	95.4±3.7	93.8±4.9	61.2±24.1
Mature, aCO_2-NN	5,760±302	97.9±1.7	95.9±2.2	89.1±5.8	83.9±8.8	43.5±33.6
Mature, eCO_2-NN	4,802±617	99.3±0.9	98.6±1.1	97.3±1.4	96.3±2.4	60.1±40.8
Total	210,423	98.7	97.9	92.7	91.1	49.4

[a] Tiller, Fill, and Mature indicate that the leaf samples were collected at the tillering, filling, and maturity stages, respectively.

[b] aCO_2-LN denotes the treatment of ambient CO_2 plus low nitrogen level fertilization; eCO_2-LN denotes the treatment of elevated CO_2 plus low nitrogen level fertilization; aCO_2-HN denotes the treatment of ambient CO_2 plus high nitrogen level fertilization; and eCO_2-HN denotes the treatment of elevated CO_2 plus high nitrogen level fertilization. Three replicates were arranged at each treatment.

The data presented are the mean values of the triplicate treatments±standard deviation.

Supplementary Figure

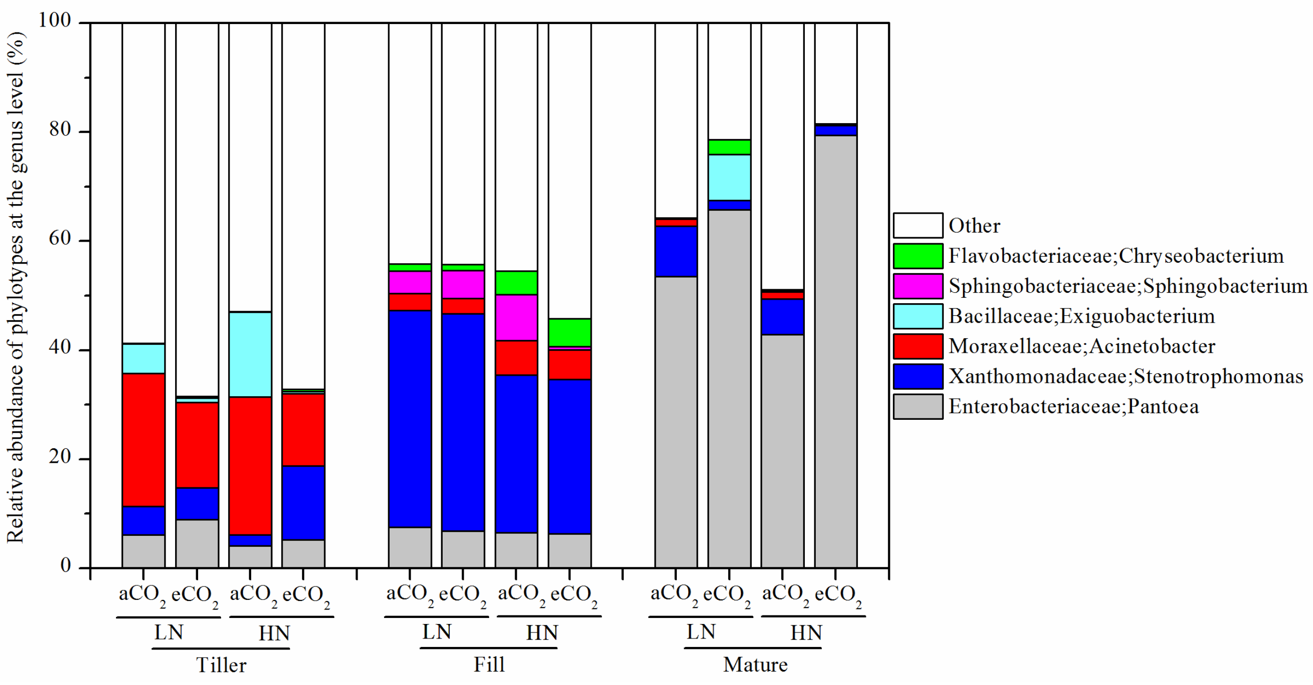

Figure S1. The relative abundance of phylotypes at the genus level. The phylotypes that had a relative abundance of >1% under at least one treatment are shown in this figure. The phylotypes are shown in the form of "family;genus".

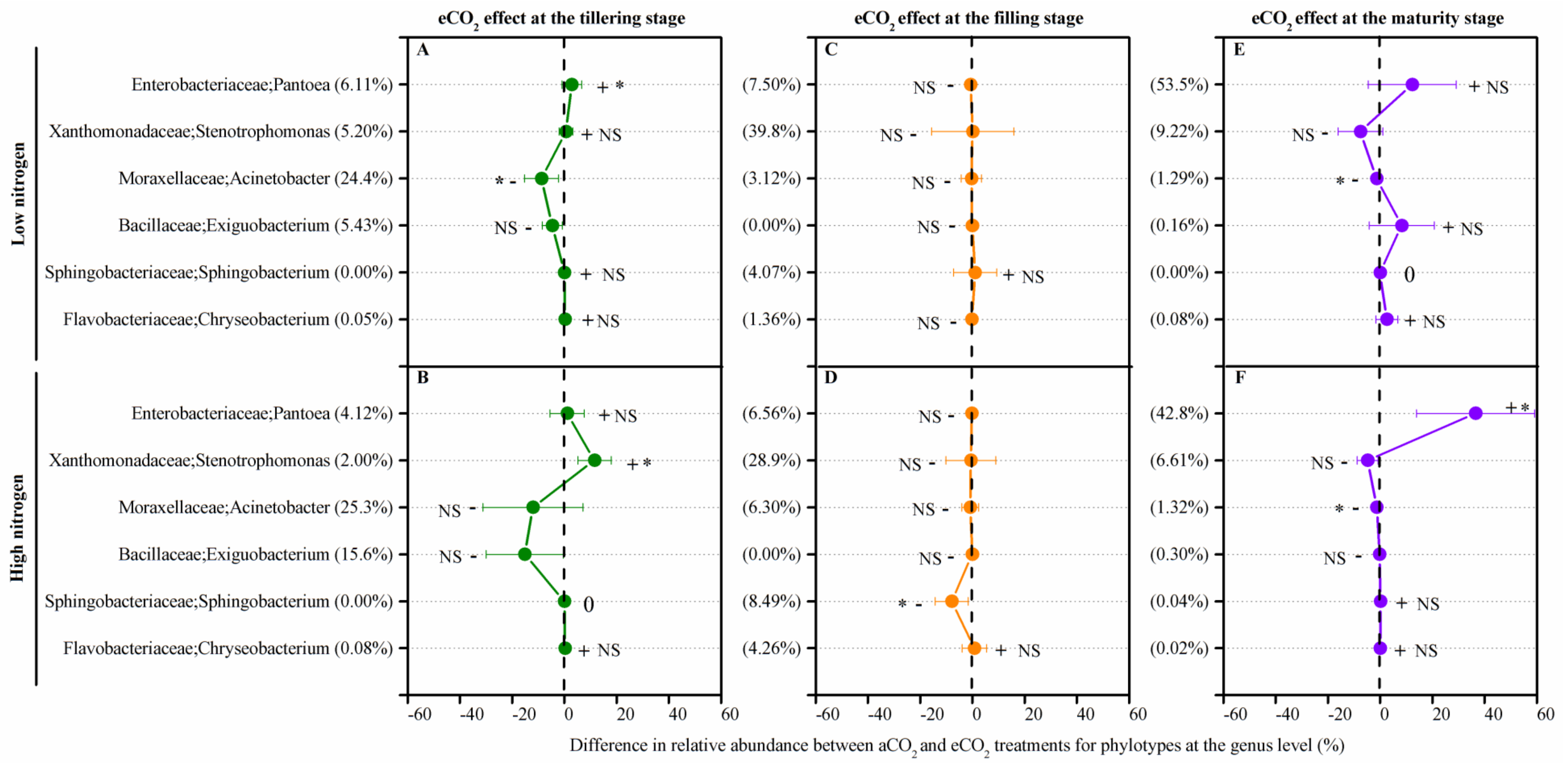

Figure S2. The effect of eCO_2 on the relative abundance of leaf endophytic bacterial phylotypes at the genus level at the tillering (A, B), filling (C, D), and maturity (E, F) stages. The net difference in relative abundance between the eCO_2 and aCO_2 was calculated as the relative abundance under eCO_2 minus the relative abundance of the phylotype under aCO_2 at each N treatment level. The percentage value in the bracket represents the relative abundance at aCO_2. The error bar denotes the standard error of the mean. The symbols “+”, “-”, and “0” indicate that the relative abundance was increased, decreased, or stable compared with the aCO_2 control treatment. The symbol “*” represents significant differences at $P<0.05$ and NS represents no significant difference ($P>0.05$). All other designations are the same as those in Table S1 or Figure S1.

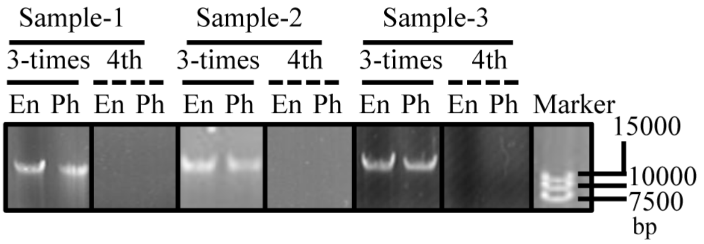

Figure S3. The electrophoresis results of microbial DNA from the leaf endosphere (En) and phyllosphere (Ph).

3-times: the DNA was extracted from the microbial cells that were collected in the initial three washing procedures.

4th: the DNA was extracted from the microbial cells that were collected in the 4th washing process.
